# Current status, breeding strategies and future prospects for managing chilli leaf curl virus disease and associated begomoviruses in Chilli (*Capsicum* spp.)

**DOI:** 10.3389/fpls.2023.1223982

**Published:** 2023-10-23

**Authors:** Manoj Kumar Nalla, Roland Schafleitner, Hanu R. Pappu, Derek W. Barchenger

**Affiliations:** ^1^ World Vegetable Center, South and Central Asia Regional Office, Hyderabad, India; ^2^ World Vegetable Center, Tainan, Taiwan; ^3^ Department of Plant Pathology, Washington State University, Pullman, WA, United States

**Keywords:** *Capsicum* spp., leaf curl virus, begomoviruses, geminiviruses, epidemiology, diversity, management

## Abstract

Chilli leaf curl virus disease caused by begomoviruses, has emerged as a major threat to global chilli production, causing severe yield losses and economic harm. Begomoviruses are a highly successful and emerging group of plant viruses that are primarily transmitted by whiteflies belonging to the *Bemisia tabaci* complex. The most effective method for mitigating chilli leaf curl virus disease losses is breeding for host resistance to *Begomovirus*. This review highlights the current situation of chilli leaf curl virus disease and associated begomoviruses in chilli production, stressing the significant issues that breeders and growers confront. In addition, the various breeding methods used to generate begomovirus resistant chilli cultivars, and also the complicated connections between the host plant, vector and the virus are discussed. This review highlights the importance of resistance breeding, emphasising the importance of multidisciplinary approaches that combine the best of traditional breeding with cutting-edge genomic technologies. subsequently, the article highlights the challenges that must be overcome in order to effectively deploy begomovirus resistant chilli varieties across diverse agroecological zones and farming systems, as well as understanding the pathogen thus providing the opportunities for improving the sustainability and profitability of chilli production.

## Introduction

**Figure 1:**
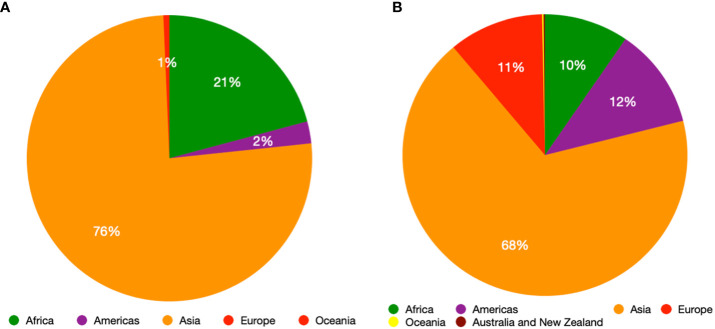
Production quantity share (Metric tonnes) of green chilli across the regions of the world **(A)** and production quantity share (Metric tonnes) of dry chilli across the regions of the world **(B)**.

Chilli (*Capsicum* spp.) is one of the oldest domesticated crops originating in the Americas (Bosland and Votava, 2012). The genus *Capsicum* comprises of about 38 species with extensive diversity in plant, flower, and fruit traits (Khoury et al., 2020). *Capsicum annuum* (L.), *C. baccatum* (L.), *Capsicum chinense* (Jacq.), *Capsicum frutescens* (L.) and *Capsicum pubescens* (Ruiz & Pav.) are the five domesticated species with *C. annuum* being the most widely grown and consumed (Bosland and Votava, 2012). Chilli pepper, comprising around 17% of the global spice trade (Ahmed et al., 2000), is an essential ingredient contributing flavor and spiciness to numerous cuisines around the world (Bosland and Votava, 2012). Chilli production and consumption have increased over the past three decades, especially for hot chilli peppers and an estimated quarter of the world’s population consumes chilli on a daily basis (Halikowski-Smith, 2015), rising from 1.4 to roughly 4.2 million tonnes of dried types and from about 14 to 38 million tonnes of fresh types. Approximately 65% of chilli is produced in Asia (FAOSTAT, 2022) (**Figure 1**) and being a high value crop (DeWitt and Bosland, 1993), chilli can have economic benefits for smallholder farmers, greatly improving family income and socioeconomic mobility (Weinberger and Lumpkin, 2007; Kahane et al., 2013).

Though chilli is considered to be a hardy plant, it is affected by several pests and diseases causing extensive losses. The past three decades have seen an increasing number of viral diseases causing considerable yield loss in several parts of the world (Suzuki and Mori, 2003; Kenyon et al., 2014b). Among the begomoviruses causing viral diseases in chilli, chilli leaf curl disease (ChiLCD), caused by Chilli leaf curl virus (ChiLCV), is the most problematic disease in the Asiatic region (Kenyon et al., 2014a; Thakur et al., 2018). Whereas, in the Americas, Pepper golden mosaic virus (PepGMV) and Pepper huasteco yellow vein virus (PHYVV) cause significant yield losses in pepper production (Devendran et al., 2022).

The primary focus of this review is directed towards discussing the Chilli pepper begomovirus diseases thereby underscoring the significant challenges that breeders and farmers are facing. In chilli, diseases caused by begomoviruses are a relatively recent problem. Pernezny et al. (2003) have reported five begomoviruses affecting chillies in the Americas and only one virus in Asia. Since then, the number of begomoviruses infecting chillies in Asia has greatly increased, with at least 29 species and a large diversity of strains reported (Kenyon et al., 2018). This increase is partly a result of more intensive investigation, but probably more importantly due to the rapid evolution and recombination between viruses, particularly under strong selection pressure.

**Figure 2:**
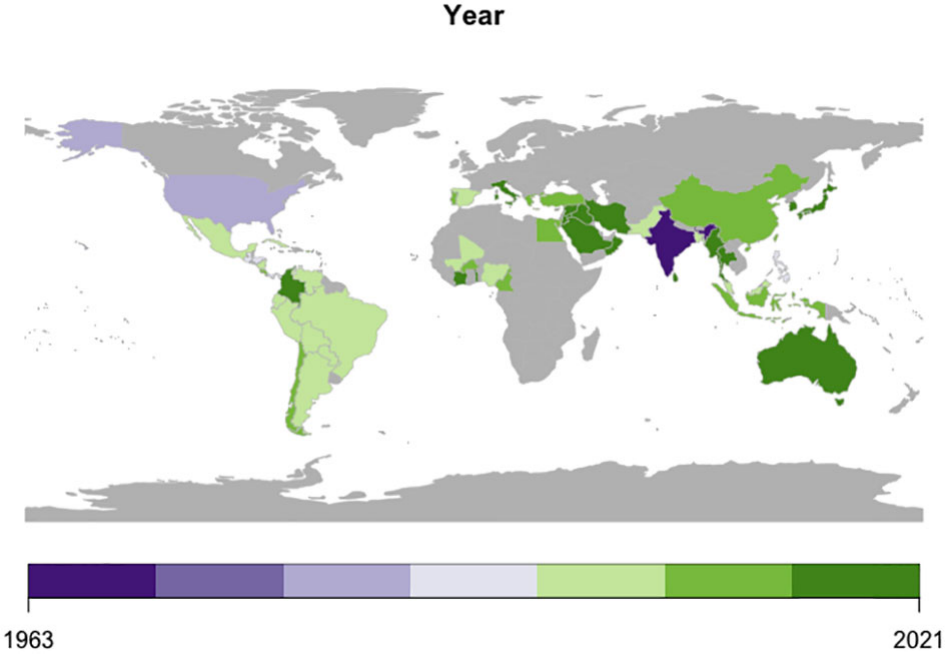
Global spread of *Begomovirus* infection in chilli.

The genomes of begomoviruses consists of either a single-stranded DNA composed of one (monopartite) or two (bipartite) components of size 2.5- 3 kb, known as DNA-A and DNA-B (Sakata et al., 2008; Zerbini et al., 2017). Begomoviruses reported in the New World (NW) regions, such as in the American regions (Latin America and North America), mostly have bipartite genomes, whereas begomoviruses reported in the Old World (OW) regions, such as Africa, Asia, Australia, and Europe, are mostly monopartite genomes with few exceptions (Devendran et al., 2022). Members of the genus *Begomovirus* infect dicotyledonous plants, are widely distributed globally (**Figure 2**), and are classified based on the presence of separately encapsulated genome components- as monopartite with a DNA-A-like component and as bipartite containing DNA-A and DNA-B (Brown et al., 2012). Generally, DNA-A encodes six open reading frames (ORFs) necessary for viral replication, transcription, activation, and encapsidation. The DNA-B component facilitates cell to cell and nucleocytoplasmic trafficking of the viral genome (Kumar et al., 2015). In monopartite species, intracellular movement is controlled by the DNA-A-like component (Hanley-Bowdoin et al., 2013). The two components of bipartite genomes share a common region, origin of replication (ORI) of approximately 200 nt (Brown et al., 2012). Begomoviruses are seen in association with satellite of ~1.3 kb circular single-stranded DNA molecules that are often necessary for symptom development in the host (Briddon et al., 2001; Saunders et al., 2004). The tendency for genetic recombination or the acquisition of extra DNA components, and the synergistic interaction among different begomoviruses have resulted in the emergence of new viruses and strains that overcome host resistance and often resulting in more severe and different disease symptoms and an expansion of the host range (Lefeuvre et al., 2007).

### Genetic recombination, mutation, synergy in Begomoviruses

Members of the genus *Begomovirus* are among the most economically damaging pathogens and pose a significant threat to the production of chilli (Senanayake et al., 2007). Begomoviruses were historically divided, based on geographical origin, into New World and Old World groups, with the New World isolates being bipartite and Old World isolates being mostly monopartite (Zhou, 2013). However, there are numerous exceptions to this classification (Melgarejo et al., 2013), and there is genetic evidence that New World isolates were present in the Old World prior to continental drift (Ha et al., 2008), making this classification arbitrary. Although chilli leaf curl virus disease has been reported to be associated with both bipartite and monopartite begomoviruses (Hussain et al., 2004; George et al., 2014), it was historically associated primarily with a complex of monopartite begomoviruses, and a diverse group of betasatellites (Kumar et al., 2015). However, in the years since this study, more and more bipartite begomoviruses have been reported in association with chilli leaf curl virus diseases (Kenyon et al., 2018).

**Figure 3:**
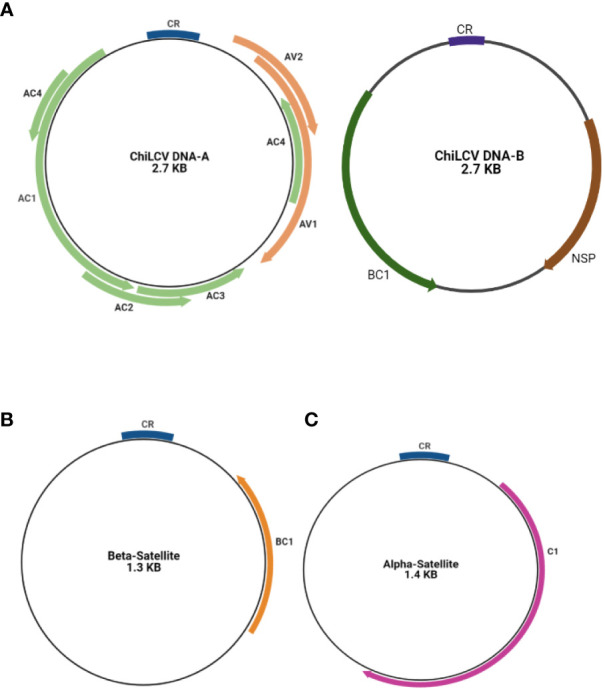
Basic genome structure of begomovirus causing chilli leaf curl virus disease **(A)** DNA-A segment and DNA-B segment **(A)** and its associated beta satellite **(B)** and alpha satellites **(C)**.

The DNA-A encodes six ORFs that encode protein for replication, encapsidation and movement AV1/V1 and AV2/V2 in sense orientation and AC1/C1, AC2/C2, AC3/C3, and AC4/C4 in the antisense orientation (Fontenelle et al., 2007; Hanley-Bowdoin et al., 2013). The AC1 encodes for a Rep (replication associated protein) and AC2 for a TrAP (transcriptional activator protein) while the protein encoded by AC3 is the Ren (replication enhancer protein) whereas the protein encoded by AC4 serves as RNA silencing suppressor. The other two ORFs are AV1 coding for a coat protein and AV2 coding for a protein whose function is unknown (Chattopadhyay et al., 2008). Similarly, the DNA-B component also contains two ORFs (BC1 and BV1). BC1 and BV1 regions contain fundamental elements that were necessary for the replication and transcription of the viral genome. NSP (Nuclear Shuttle Protein) aids in the movement of viral genetic material within the nucleus of the host cell. C1 is a Replication-Associated Protein that is essential for viral replication. The Common Region (CR) contains regulatory components that are required for replication, recombination, and gene expression control. Understanding these areas and their functions is essential. (**Figure 3**) (Sahu and Mishra, 2021; Shingote et al., 2022). Various plant cellular and physiological pathways that are intrinsically maneuvered by geminiviruses for the spread and establishment of infection in the plant (Sahu et al., 2014). DNA-A encoding proteins are responsible for replication, encapsidation and vector transmission while DNA-B encoding proteins have movement related functions (Shahid et al., 2019).

Mutation, recombination and pseudo-recombination are the major drivers of evolution and genetic variation of plant viruses (Juarez et al., 2019). Genetic variation favors evolutionary potential and adaptation of viral populations with novel pathogenic attributes to a changing environment (Lima et al., 2017) and to host resistance (Escriu, 2017). In the evolution of geminiviruses, recombination has played an especially impactful role (Varma and Malathi, 2003; Lefeuvre and Moriones, 2015). Due to high mutation and recombination rates, genetic variability of begomoviruses increases at a rapid and significant rate (Fiallo and Navas, 2023). Kumar et al. (2015) suggested that the recombinant begomoviruses and betasatellites were the major factors for the emergence of chilli leaf curl virus disease epidemics in India. Infectious recombinants or pseudo recombinants of begomoviruses can emerge during multiple infections (Pita et al., 2001; Mendez-Lozano et al., 2003). Chakraborty et al. (2008) reported the formation of a viable super virulent pseudorecombinant of tomato leaf curl New Delhi virus (ToLCNDV) (DNA-A) and tomato leaf curl Gujarat virus (ToLCGV) (DNA-B), which caused severe leaf curl disease in tomato. Recombination analysis indicated that the chilli leaf curl Palampur virus (ChiLCPaV) strain had likely descended from a sequence that arose through interspecific recombination between tomato leaf curl Karnataka virus (ToLCKV) and croton yellow vein mosaic virus (CYVMV) (Kumar et al., 2011). The A-rich region and satellite conserved regions (SCR) were reported as hot spots for recombination among chilli-infecting beta-satellites (Pan et al., 2020). It has been suggested that the high rate of recombination events of the DNA-A component and their respective betasatellites are the major reasons for the occurrence of ChiLCV in the previous non-host crops such as Cape Daisy (*Osteospermum fruticosum*) (Mishra et al., 2020a), *Amaranthus* spp. (George et al., 2014), *Mentha spicata* (Saeed et al., 2014), *Petunia* spp. (Al-Shihi et al., 2014) and *Mirabilis jalapa* (Jaidi et al., 2017).

Plant viruses co-infecting the same host plant can interact in either in a synergistic or an antagonistic way (Rentería-Canett et al., 2011). Mixed infections faciliatate recombination, which could lead to the appearance of more severe strains or new *Begomovirus* species (Wahyono et al., 2023). Previous studies reported mixed infection of leaf curl-causing new and more virulent viruses in chilli (Singh et al., 2016; Mishra et al., 2020a). The preference of whiteflies for multiple hosts and transmission of multiple viruses simultaneously favor these mixed infections. Synergistic interaction within different begomoviruses strains during multiple infections was shown to result in increased viral DNA accumulation in the infected host plants along with the tendency to suppress plant defense mechanisms (Burgyan and Havelda, 2011; Caracuel et al., 2012). The synergistic relationships among begomoviruses were shown to result in a permissive cellular environment in the resistant chilli plants, which lead to the breakdown of host resistance (Singh et al., 2016). Synergistic interactions have been reported by Singh et al. (2016) where an association of four viral genomic components in symptomatic chilli cultivars resulted in increased viral DNA accumulation and severe symptoms. Similarly, Rentería-Canett et al. (2011) reported synergistic interaction of two begomoviruses (pepper golden mosaic virus (PepGMV) DNA-A, and DNA-A and DNA-B of pepper huasteco yellow vein virus (PHYVV) resulting in a marked increased disease severity in *N*. *benthamiana*, chilli, and tomato plants. In contrast, antagonistic interactions were also found in chilli plants co-infected with pepper huasteco virus (PHV) and PepGMV (Mendez-Lozano et al., 2003). Alves-Júnior et al. (2009) demonstrated that the reduced titers of viruses in tomato infected by tomato yellow spot virus (ToYSV) and tomato rugose mosaic virus (ToRMV) may be due to the antagonistic negative interference between two begomoviruses even though the symptoms expressed were more severe in comparison with single infections. More intensive studies of the synergistic and antagonistic interactions among the viruses could provide important insights into viral pathogenesis and evolution.

High rates of mutations and frequent recombination’s are the major cause for the rapid evolution and genetic variability in begomovirus populations (Lima et al., 2013). Mutations in the coat protein (CP) may play a key role in both the adaptation of begomoviruses to the changing vector populations and the evolution of begomoviruses (Pan et al., 2020). Vector transmission also gets affected by the genetic variation in the begomoviruses. Mutation in the CP gene may lead to variations in the transmissibility by a given whitefly species (Caciagli et al., 2009). Pan et al., 2020, described that the whitefly transmission characteristics of squash leaf curl China virus (SLCCNV) is significantly modified due to a single mutation in the CP. Similarly, Noris et al. (1998) documented that the double mutation in the CP of tomato leaf curl Sardinia virus (TLCSaV) resulted in the loss of whitefly transmissibility. However, the adaptation of viruses to insect vectors after genetic variation due to mutation and recombination is unclear. Viral mutations, recombination and genetic reassortment constitute the biggest threat to chilli cultivation in terms of the breakdown of host plant resistance. Knowledge about the existence and frequency of recombination in a viral population could help understand the extent to which genes are exchanged potentially leading to the emergence of new virus variants.

### Symptoms associated with Chilli leaf curl virus disease

Viral disease epidemics are affected by various factors such as insect vectors, viral species, and the environment (Mahatma et al., 2016). In tropical and subtropical regions, chilli faces severe losses, up to 100%, due to chilli leaf curl virus disease (Chattopadhyay et al., 2008; Varma et al., 2011; Senanayake et al., 2012; Srivastava et al., 2017; Kumar, 2019; Mandal et al., 2017). The emergence of new virus strains and the spread of whitefly ‘B’ biotype, lack of resistance or breakdown of host resistance, ineffective insecticides, increased vector host range, are major factors that influence the chilli leaf curl virus disease outbreaks and crop loss. It is generally agreed that seed transmission of geminiviruses does not occur. However, Fadhila et al. (2020) reported that the pepper yellow leaf curl Indonesia virus (PepYLCIV) is seed-transmissible. The molecular analysis revealed that embryos and seedlings grown from PepYLCIV infected chilli seed collected from various locations indicated 25-67% PepYLCIV DNA-A and 50-100% PepYLCIV DNA-B. The possibility of seed transmission needs to be further investigated, because other factors could contribute to this finding.

Infection is the outcome of complex tripartite interaction among host plants, insect vectors and infecting viruses (Sun et al., 2017). Begomoviruses are transmitted by whiteflies in a persistent and circulative manner. The interaction between begomoviruses and the whitefly vector is well understood. Jasmonic acid (JA) plays a role in host resistance to whitefly and begomovirus infection has been shown to reduce transcription of some JA-responsive genes, enhancing vector survival and reproduction (Li et al., 2019). It has been reported that the begomovirus virulence factors suppress terpene production, reducing host resistance to the whitefly vector (Li et al., 2014), thereby increasing the spread of the virus. Management of begomoviruses has been based primarily on insecticides against the whitefly vector. However, the use of insecticides has been found to be only partially effective, costly for producers, and represents a hazard to farmers, consumers, and the environment (Borah and Das, 2012), while limiting export potential because of the presence of pesticide residues. Furthermore, using insecticides to manage the vector is often ineffective because transmission of the virus occurs during the vector’s probing of the plant surface, prior to feeding, and before the insecticides can take effect (Kenyon et al., 2014a).

It is likely that chilli leaf curl virus diease existed in as early as 1963 in India; however, only reports of symptoms exist from this time (Mishra et al., 1963) and associaton with begomoviruses is more recent. Since 2005 the severity and occurrence of the begomovirus in chilli has significantly increased worldwide (Kenyon et al., 2014a). The diseases elicited by begomoviruses in chilli can be characterized into three broad categories of symptoms; vein yellowing, yellow mosaic, and leaf curl. Apart from leaf anatomy damage, the virus alters the plant physiological functions and fruit production resulting in axillary buds turning into leaf clusters, failing to bear any fruit, plant stunting, and eventually leading to plant death and complete crop loss. A wide range of symptoms are associated with chilli leaf curl virus infection and includes leaf mosaic, leaf curling, chlorosis, rolling, crinkling, cupping, puckering, enations, blistering, petiole bending and twisting, crowding of leaves, vein clearing, plant stunting and reduced fruit number and size (Stenger et al., 1990; Torres-Pacheco et al., 1996; Khan et al., 2006; Senanayake et al., 2007; Taibangnganbi et al., 2017; Chiemsombat et al., 2018; Manisha et al., 2020; Hernández-Verdugo et al., 2001; Fadhila et al., 2020).

Incidence and severity of the disease under natural field conditions is influenced by various external factors. Symptoms can be affected by environmental factors such as soil fertility and microclimate around plants, age of the plant and host genetic makeup (Matthews, 1991) in addition to the species or strain of the virus. Increased severity of chilli leaf curl virus associated symptoms has also been observed in the presence of cognate betasatellites molecules (Kumar et al., 2016). Betasatellites were found to be a prerequisite for the induction of severe leaf curl symptoms in *Capsicum* spp. although the viral genomic DNA-A and-B contain open reading frames (ORFs) known to cause infection (Chattopadhyay et al., 2008; Ruhel and Chakraborty, 2019). Typical symptoms of chilli leaf curl virus have been observed only when both the viral genome and satellite DNA were present (Chattopadhyay et al., 2008). Kumar et al. (2011) provided further evidence for the role of betasatellite molecules in the induction of leaf curl symptom in *C. frutescens*. When *C. frutescens* plants were agroinoculated with infectious clones that included both the betasatellite (1.7-mer) and partial tandem repeats of the viral genome (1.9-mer), the characteristic ChiLCV symptoms such as leaf curling and stunting appeared, but when the viral genome alone was used, no symptoms of leaf curling appeared. Furthermore, synergistic interactions among different *Begomovirus* species could lead to increased symptom severity and new and diverse symptoms, in addition to the new viral species with an expanded host range.

### The whitefly vector

Whitefly (*Bemisia tabaci* Genn.; Hemiptera: Aleyrodidae) is a polyphagous insect that feeds on over 361 plant species from 89 families (Li et al., 2011). Recently, it has been reported that the broad host range of whitefly could to be due to the presence of a plant-derived phenolic glucoside malonyltransferase gene, *BtPMaT1*, which enables whiteflies to neutralize phenolic glucosides, a toxin produced by plants as a defense mechanism (Xia et al., 2021). Interestingly, the horizontal transfer of the *BtPMaT1* gene from plants to whitefly is predicted to have been mediated by a viral species. The primary damage caused by whitefly, from a phytopathological view, is their role as vectors for plant viruses. However, as sucking pests, their feeding also causes direct damage to the plant and can result in a reduction in photosynthetic capacity. Furthermore, their feeding nymphs excrete honeydew which promotes sooty mold that interferes with photosynthetic activity of plants. Geminiviruses and whiteflies have been interacting for millennia (Czosnek et al., 2001). Whiteflies ingest virus through their stylets while feeding on the phloem of infected plants (acquisition access period (AAP)) and ingest the virus with saliva into the phloem of other plants (inoculation access period (IAP). When whiteflies suck the phloem sap from infected tissue, virions reach the insect midgut via the stylet and eventually reach the salivary glands, from where they are transmitted to new plants during feeding or probing (Sinisterra et al., 2005; Wei et al., 2014). Along with DNA A and B, circular beta-satellites (Tabein et al., 2013), and delta-satellites (Hassan et al., 2016) are transmitted by whiteflies in the presence of helper viruses. Whiteflies transmit begomoviruses in a persistent manner and will be transmitted only after the incubation period of hours to days (Ghanim et al., 2001). The AAP and IAP required for adult whiteflies have been reported for many *Begomovirus* species (Radhakrishnan et al., 2004; Hidayat and Rahmayani, 2007). Studies on the virus-vector relationships revealed that the AAP and IAP for begomoviruses range from 30 to 210 and 5 to 60 min, respectively (Senanayake et al., 2012). The transmission efficiency usually differs among *Begomovirus* species however, for artificial inoculation, the IAP used is considerably longer than required to ensure successful transmission (Barchenger et al., 2019).

The recent unprecedented upsurge of whitefly populations has been identified as a major contributor to the chilli leaf curl virus epidemics in recent years (Kumar et al., 2015; Padhi et al., 2017). The rapid spread of begomoviruses infecting chilli has been associated with an expansion of polyphagous whitefly B-biotype that are able to breed twice faster thatn non-B biotype (Wang et al., 2023). Plant viruses can produce direct and plant-mediated indirect effects on their insect vectors, modifying their life cycle and behaviour (Moreno-Delafuente et al., 2013). The B-biotype has been reported to have an increased fecundity in tomato leaf curl china virus (TYLCCNV) infected plants (Luan et al., 2013). Further, it was hypothesized that the virus could induce behavioural change of the vector as well as plant biochemical composition, increasing whitefly spread (Brown, 2000). Co-evolution between the viral capsid protein and whiteflies favors the rapid spread and increased host range of begomoviruses across the globe. Several studies have reported that begomoviruses associated with certain crops (such as chilli or tomato) can be transmitted to new host species through whitefly vectors (Senanayake et al., 2012; Kushwaha et al., 2015). In contrast, there seems to be some level of host specificity that occurs among begomoviruses. It has been reported that tomato yellow leaf curl virus (TYLCV) is able to infect and replicate in chilli and sweet pepper; however, chilli is typically asymptomatic or develop mild symptoms when various strains and inoculation techniques were used (Morilla et al., 2003; Kil et al., 2016).

### Detection and diagnosis

Diagnosis of ChLCV based on symptoms alone is not definitive or reliable. Mixed or co-infections of multiple species of begomoviruses as well as other viruses, particularly members of the genera *Potyvirus* and *Cucumovirus* are common and these mixed infections confound accurate diagnosis (Naresh et al., 2016). Although serological techniques like dot blot hybridization and enzyme-linked immunosorbent assay (ELISA) have been used in the past to identify begomovirus, they are less accurate than molecular techniques. A laboratory test based on molecular detection techniques such as polymerase chain reaction (PCR) and the use of species-specific primers; partial nucleotide sequencing of the viral genome, whole genome amplication by rolling circle amplification (RCA) followed by sequencing (Khan et al., 2006; Senanayake et al., 2007; Chattopadhyay et al., 2008). Recombinase polymerase amplification (RPA) and loop-mediated isothermal amplification (LAMP) can also be utilized for field-based diagnosis of begomoviruses because they don’t need thermocycling equipment and can be carried out on portable devices. Development of on-site adaptable RPA-based rapid tests would be valuable in surveys as well for screening breeding materials for virus resitance, and for epidemiological and genetic diversity studies. Viruses can be detected and diagnosed quickly, easily, and accurately using isothermal-based assays. LAMP and RPA are two of the most popular isothermal amplification assays as they don’t need thermocycling equipment and can be carried out on portable devices. Isothermal amplification kits have been commercially available and are currently being used for the detection of begomoviruses in a wide range of crops. The choice of diagnostic method may depend on factors such as cost, availability of equipment and expertise.

### The role of the satellite molecules in *Begomoviruses* pathogenesis

Generally, satellite RNAs have been widely found to be associated with RNA plant viruses (Simon et al., 2004), it was not until 1997 that the first DNA satellite was identified to be associated with the monopartite begomoviruses, tomato leaf curl virus (ToLCV) (Dry et al., 1997). Begomoviruses are primarily associated with two classes of ssDNA satellite molecules, known as alphasatellites and betasatellites. Alphasatellites are capable of self-replicating in their hosts, but require helper begomoviruses for movement in plants and insect transmission (Zhou, 2013). Betasatellites are generally associated with many monopartite begomoviruses and are essential for infection and the induction of typical disease symptoms (Jose and Usha, 2003; Saunders et al., 2004). Similar to alphasatellites, betasatellites also depend on begomoviruses for cell-to-cell and systemic spread throughout the host, for encapsidation, and for transmission to new host plants via whitefly vectors. Begomovirus-satellite complexes infect a wide range of plants within at least 37 genera and 17 different families (Zhou, 2013).

Betasatellites typically contain a satellite conserved region (SCR), an adenine-rich region, and a βC1 ORF (Shingote et al., 2022 Single-stranded satellite DNA has been reported to be associated with both bipartite and monopartite begomoviruses (Hussain et al., 2004; George et al., 2014). Betasatellites associated with monopartite viruses were around half the size of their helper begomoviruses genome and are essential to induce typical symptoms of virus diseases in their hosts (Briddon et al., 2002). Chattopadhyay et al. (2008) reported for chilli leaf curl disease (ChiLCD) caused by a complex mixed virus particle consisting of the virus variant of monopartite chilli leaf curl virus (ChiLCV) and a betasatellite variant of tomato leaf curl Bangladesh virus (ToLCBDB) (Chattopadhyay et al., 2008). The *Begomovirus* genus betasatellite molecules are important determinants of pathogenicity for most monopartite begomoviruses in many economically important crops (Yang et al., 2011). For replication, encapsidation and cell-to-cell motion, the beta satellite molecule relies on the helper virus (Briddon and Stanley, 2006). Betasatellites encodes a 13.5 kDa protein named βC1, a suppressor of gene silencing essential (Transcriptional gene silencing (TGS) and PTGS (Post-transcriptional gene silencing) (Cui et al., 2005; Li et al., 2014) contributes to pathogenesis (Eini et al., 2009; Badar et al., 2020), affect JA-responsive gene (Yang et al., 2011) and capable of functionally replacing the DNA-B encoded movement protein (Patil and Fauquet, 2010). Additionally, betasatellites also contribute to greater accumulation of viral DNA in the infected tissues (Sivalingam and Varma, 2012). Recently, Kumar et al. (2015) illustrated the need for beta-satellites to develop extreme chilli leaf curl disease. They observed mild symptoms in the chilli plants when inoculated with DNA-A like sequences, but in the presence of cognate betasatellites along with the accumulation of viral DNA, the severity of the symptoms was increased. Symptom enhancement due to betasatellites is not restricted to monopartite begomoviruses; the interaction of bipartite begomoviruses and betasatellite is also a cause.

Alphasatellites are 1.3-1.4kb sized DNA molecules that are coupled with begomoviruses and betasatellite complexes (Briddon et al., 2004). Though their role is obscure, they were considered as a class of self-replicating circular ssDNA satellite like molecules that require helper viruses for their intra and intercellular movement, encapsidation, reduction in betasatellites accumulation and doesn’t have any role in symptom induction (Nawaz-ul-Rehman et al., 2010; Idris et al., 2011; Xie et al., 2002). Alpha satellites encode their own nanovirus-like replication initiator protein called alpha Rep (Saunders and Stanley, 1999; Badar et al., 2020). By overcoming host defense by RNA silencing, alphasatellites also play an imperative role in begomoviruses epidemiology (Nawaz-ul-Rehman et al., 2010). Deltasatellites are small (~0.7kb) noncoding DNA satellites associated with begomoviruses that diminish the accumulation of the helper begomovirus in the plant, seldom modify the begomoviruses symptoms but do not encode for any protein. While satellite DNA molecules depend on their helper begomoviruses for cell-to-cell movement and systemic spread throughout the plant, encapsidation, and transmission to new host plants by insect vectors (Xie et al., 2002), they can play a critically important role in the disease severity and breakdown of host resistance. There is a need to further study satellite DNA molecules and to understand their evolution and diversity in order to effectively breed for host resistance.

### Genetic diversity in chilli leaf curl virus and associated beta satellites

**Figure 4:**
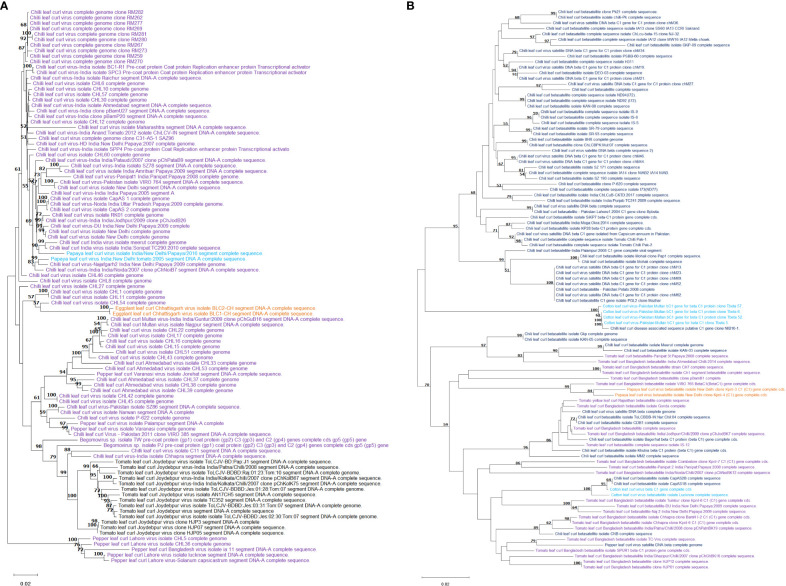
Phylogenetic analysis of begomovirus **(A)** and betasatellite **(B)** associated with chilli leaf curl virus in chilli and closely related leaf curl viruses retrieved from NCBI GenBank. The sequences were aligned using MUSCLE and the tree constructed in MEGA by using Neighbor-Joining method following maximum likelihood criterion with 1000 bootstrap. The scale bar represents the rate of nucleotide substitutions per site.

The complete genome sequences of 83 isolates of begomoviruses associated with chilli leaf curl virus disease along with closely related begomoviruses associated with papaya, eggplant and tomato leaf curl diseases retrieved from the NCBI GenBank when subjected to phylogenetic analysis, formed 2 distinct clusters (**Figure 4A**), the isolates of chilli leaf curl virus clustered together along with papaya leaf curl virus and eggplant leaf curl virus. Isolates of tomato leaf curl virus are grouped in a separate subcluster. However, pepper leaf curl virus isolates from Lahore (Pakistan), Bangladesh, and Lucknow (India) are distinctly separated out into a cluster which represents more genetic distinctness from the rest of the leaf curl viruses analyzed. Similarly, in a separate phylogenetic tree (**Figure 4B**) for analysis of genetic relatedness in beta satellite segment associated with chilli leaf curl virus along with tomato-, papaya- and cotton leaf curl virus revealed that beta satellite molecules associated with ChiLCV virus are similar in their genetic makeup with other leaf curl viruses infecting tomato (*Solanum lycopersicum* L.), papaya (*Carica papaya* L.) and cotton (*Gossypium* spp. L).

### Managing chilli leaf curl virus disease: progress and challenges

Management of begomoviruses has been based primarily on insecticides against the whitefly vector. However, the use of insecticides has been found to be only partially effective, costly for producers, and represents a hazard to farmers, consumers, and the environment (Borah and Das, 2012). Insecticides to manage the vector are often ineffective because transmission of the virus occurs during the vector’s probing of the plant surface, prior to feeding, and before the insecticides can take effect (Kenyon et al., 2014a). Similar to *Begomovirus*, whiteflies often evolve into new biotypes and can become tolerant to commonly used insecticides (Naveen et al., 2017). Whiteflies have shown resistance to more than 40 active ingredients of insecticides (Whalon et al., 2013).

The currently available strategies to manage begomovirus include host plant resistance (although tolerance might be a more accurate description of the best commercially available cultivars) (Barchenger et al., 2019), insect resistance (although no sources of white fly resistance in *Capsicum* spp. have been reported), pathogen derived resistance, insecticide application (Bragard et al., 2013) and the much less practised changes in cropping systems and sanitation. Jabłońska-Sabuka et al. (2015) utilized a mathematical model to predict epidemics and understand the global spread of begomovirus and found that intensive farming and breeding partially resistant cultivars were the major triggers for aggressive virus adaptability through increased rate of mutation.

When it comes to managing plant viral infections, smallholder farmers in Asia and Africa confront a number of obstacles. One of the most significant obstacles is a lack of access to information and resources, as well as limited access to and high cost of pesticides. Overuse of pesticides may result in the development of resistance in pest populations. Another barrier is a lack of understanding and awareness of plant viral infections. Smallholder farmers may be unaware of the indications of viral illnesses and may lack knowledge of how to adopt good disease management practices. In addition to these, the problem of plant viral infections is being made worse by climate change. The distribution and abundance of viral vectors, as well as the susceptibility of host plants to infection, can be impacted by changes in temperature and rainfall patterns. Smallholder farmers may find it more difficult to adequately manage viral infections as a result.

For a management program that is to be widely adopted by farmers – it should be simple, inexpensive, and practical. One of the most important tools for managing plant viral diseases in smallholder farms is integrated pest management (IPM). IPM is a holistic approach to pest control that emphasizes the use of cultural, biological, and chemical control measures to manage pests and diseases in a sustainable and environmentally friendly way. IPM can be particularly useful for managing insect-transmitted viruses, as it can help reduce the vector population. Additionally, IPM can help to prevent the development of resistance in pest populations, which can make pest control more effective in the long run.

Host plant resistance and use of resistant cultivars, combined with other production practices, forms the central component of a successful IPM program for reducing the impact of viral diseases. It has been estimated that farmers that adopt low (one or a few strategies), medium, and high (multiple strategies) integrated management strategies for begomovirus could improve incomes by 17, 26, and 80%, respectively (Swaminathan et al., 2016).

It is known that whiteflies can travel several kilometers and are semi-persistent and there is a direct relationship between vector level in neighboring fields and virus incidence in test fields (Aritua et al., 1999). Therefore, old infested plantings can be a source of whitefly and virus inoculum and need to be completely removed prior to new plantings. In addition, weeds in and around the field can serve as whitefly reservoirs. In Madhya Pradesh and Andhra Pradesh states of India, for example, chilli and cotton are grown at the same time or in rotation, and cotton is a whitefly host and could serve as a reservoir. Melons grown in close vicinity to a cotton crop was found to significantly increase whitefly incidence (Ellsworth and Martinez-Carrillo, 2001), but no reports on whitefly incidence of chilli grown near to cotton have been published. The use of host-free periods can be effective in reducing whitefly populations and could be more effective when host-free periods are combined with insecticide use during production season (Ellsworth and Martinez-Carrillo, 2001). It has also been demonstrated that plant spacing can play a major role in begomovirus incidence in cassava, but no reports in chilli have been published. Similarly, it has been found that modifying the production system can limit whitefly colonization. Although there are no reports on chilli, intercropping cucumber, tomato, or squash with maize resulted in significantly lower whitefly and begomovirus incidence (Abd-Rabou and Simmons, 2012). The use of cucurbit trap crops reduced whitefly and tomato yellow leaf curl virus (TYLCV) incidence in the Southern USA; however, trap crops alone are not sufficient to keep the vector populations below the action threshold and must be combined with other management strategies.

In the present context of seemingly constant emergence of new begomoviruses, a clear understanding of the virus/whitefly vector/host plant interrelationships through epidemiological, phylogenetic approaches are needed. Identification and deployment of host plant resistance can be an effective and durable strategy against whitefly damage. Whitefly resistance may provide an important contribution in limiting losses associated with begomovirus, and whitefly tolerant accessions have been reported (Firdaus et al., 2011; Ballina et al., 2013; Latournerie et al., 2015; Rajput et al., 2017; Jeevanandham et al., 2018; Pantoja et al., 2018; Yadav et al., 2020). However, there are still no cultivars with tolerance or resistance and there is a need to screen chilli accessions for resistance to whitefly along with begomovirus for durable chilli leaf curl virus resistance.

### Resistance sources

Resistant cultivars offer sustainable management of viral diseases of plants. There is a constant need to identify the new sources of resistance to counteract the rapidly evolving begomovirus in chilli, making durable host plant resistance a crucial output of successful breeding programs. Disease resistance screening for leaf curl virus disease began in the late 1960s mostly under open field conditions (Sharma and Singh, 1985; Tewari and Viswanath, 1986). The material screened comprised locally available cultivars, lines of the domesticated *Capsicum* species and then progressed to related and wild species. For tomato, resistant sources for begomovirus have been found in the wild species of *S*. *chilense* (Gill et al., 2019), *S*. *habrochaites* (Yang et al., 2014), and *S*. *peruvianum* (Hutton et al., 2012); however, for chilli the use of wild species in breeding has been extremely limited (Barchenger and Bosland, 2019). The wild species of *Capsicum* offer an almost completely untapped reservoir of variability, which could be exploited to identify sources of resistance to begomovirus (Barchenger et al., 2019). There have been numerous reports of sources of resistance to various chilli-infecting *Begomovirus* species (**Table 1** **).** Some have suggested resistant/tolerant sources against pepper leaf curl virus (*PepLCV*) found more commonly in non- *C*. *annuum* than in *C*. *annuum* accessions (Kumar et al., 2006; Rai et al., 2010; Kumar et al., 2011; Anandhi and Khader, 2011). Others have described begomovirus resistant sources in *C*. *annuum* accessions (Kumar et al., 2006; Kenyon et al., 2014a; Srivastava et al., 2015; Singh et al., 2016; Srivastava et al., 2017; Barchenger et al., 2019) (**Table 1**). Chilli leaf curl virus resistant cultivars are rarely developed by public sector researchers, with the exception of the hybrid CH-27 (Dhaliwal et al., 2015). Most of the tolerant or partially resistant cultivars on the market today were released by the private sector. Even with continuous scientific efforts, there is a dire need for systematic screening to identify sources of the ever-evolving species of begomovirus and to develop new resistant cultivars with good horticultural traits. In the current scenario the emergence of new strains, new whitefly biotypes, a strong base of plant genetic resources is the prerequisite for the chilli leaf curl virus resistance breeding program.

**Table 1:** Available sources of resistance in *Capsicum* spp. to various members of *Begomovirus*.

Source	Inoculation method	Viral species	Reference
Puri Red, Puri Orange	Open field screening	NR^z^	Mishra et al., 1963
Pusa Jwala (NP-46A X Puri Red)	Open field screening	NR	Tewari and Ramanujam, 1974
Surjamani, Perennial, S 118, S 114	Open field screening	NR	Sooch et al., 1976
Pant C-1, Pant C-2	Open field screening	NR	Mathai et al., 1977
Cross 218, EC 121490, IC 18253, IC 18885, JCA 196, Pant C-I	Open field screening	NR	Bhalla et al., 1983
CA-960, G-4, Jwala	Open field screening	NR	Dhanju, 1983
Lorai, Longi, Pant C-I, Perennial, S 5-4, S 20-1, S 41-1, S 118-2	Open field screening	NR	Sharma and Singh, 1985
Pusa Jwala, Delhi Local, Sel 38-2-1, Sel 94-4-9-3, Sel 101-2-33	Open field screening	NR	Tewari and Viswanath, 1986
JCA 196, JCA 218, JCA 248, NP-46-A, Pant C-I, Pusa Jwala	Open field screening	NR	Brar et al., 1989
Bangla Green (BG-1), CH-1, Indonesian Selection, Laichi-1, Laichi-2, Lorai, LS-l, MF41-1, MS- 13, Pant C-I, Perennial, Punjab Lal, S 20-1, Surjamani	Open field screening	NR	Singh and Kaur, 1990
Pusa Jwala, SuryaMukhi and Loungi	Open field screening	NR	Kumar et al., 1999
PBC67	Open field screening	NR	Alegbejo, 1999
Phule Sai (moderately resistant)	Open field screening	NR	Jadhav et al., 2000
KSDA-210-10, LCA-305, LCA-324 and Hissar Vijay	Open field screening	NR	Kotreshe, 2002
*C. annuum* var. *angulosum*	Open field screening	NR	Singh and Singh, 1989
*C. chinense* accession BG-3821	Particle bombardment technique	PepGMV	Anaya-Lopez et al., 2003
Alampady local-1, Nayyattinkara local, Kottiyan local, Haripuram local, Pant C-1, Chandera local, Mangalapuram local and Kotti Kulam (Tolerant)	Open field screening	NR	Jose et al., 2003
GKC-29, BS-35, EC-497636	Open field screening followed by grafting	NR	Kumar et al., 2006
HC-28 and HC-449 (Resistant, NCH (Moderately resistant)	Open field screening	NR	Mali et al., 2006
Surajmukhi, Japani Loungi, Pant Chilli-1, Pusa Jwala and PBC473	Open field screening	NR	Awasthi and Kumar, 2008
Punjab Sindhuri and Punjab Tej (moderately tolerant)	Open field screening and artificial inoculation by viruliferous whitefly	NR	Dhaliwal et al., 2013
BS-35, GKC-29, Bhut Jolokia	Artificial inoculation by viruliferous whitefly	ChiLCV	Rai et al., 2014
PBC143, PBC144, PBC149, PBC495, VI012005, VI012907 (PI 159236),	Artificial inoculation by viruliferous whitefly	TYLCThV	Kenyon et al., 2014a
CH-27	Open field screening and artificial inoculation by viruliferous whitefly	—	Dhaliwal et al., 2015
Saurian 2010, Perennial and Japani Loungi	Artificial inoculation by viruliferous whitefly	—	Ahmad et al., 2016
Hyb3(2)-3 and Hyb3(2)-2 moderately resistant	Open field screening	NR	Yonzone et al., 2016
DLS-Sel-10, WBC-Sel-5, PBC142,	Open field screening and artificial screening by viruliferous whitefly	NR	Srivastava et al., 2017:
DLS-Sel-10	Artificial screening by viruliferous whitefly	NR	Manisha et al., 2020
BJ 001 (symptomless)	Open field screening	NR	Adluri et al., 2017
Hybrid 46 and Hot Queen	Open field screening	NR	Hussain et al., 2017
13/CHVar-1 and 13/CHVar-2-Resistant	Open field screening	NR	Padhi et al., 2017
Kumarapuram-I (A-50)	Open field screening	NR	Srinivas and Thomas, 2018
S 343, SL475, SL476	Artificial screening by viruliferous whitefly	ToLCJoV	Thakur et al., 2019
9852-123	Augmented inoculation by viruliferous whitefly	PepYLCThV	Barchenger et al., 2019
Sel-3, Sel-4, CHIVAR 1	Open field screening, artificial screening by viruliferous whitefly and graft inoculation	NR	Vijeth et al., 2020
CHUH-4	Open field screening	NR	Mondal et al., 2013
Perintis (BaPep-5)	Graft inoculation and natural screening	PepYLCIV and PepYLCAV	Koeda et al. (2021),
IHR4517, IHR4615, and IHR4630	Artificial screening by viruliferous whiteflies	ChiLCV-Rai	Yadav et al., 2022
Violet Mulak, Arka Lohit and Phule Jyothi	Open field screening and Artificial screening	ChiLCV-Rai	Sujisha (2023)

### Biochemical and morphological basis of resistance

Plants have biochemical defense mechanisms to protect them from insect pests. Morphological barriers, such as trichome type and density, and associated compounds such as acyl sugars also play a role in defense against insects. A plant that is highly resistant to whiteflies is also protected against whitefly transmitted viruses (Broekgaarden et al., 2007). Occurrence and population dynamics of the vector whitefly and the weather conditions in the agroecosystem are responsible for the differential response of genotypes to chilli leaf curl virus incidence and symptom expression (Kaushik and Dhaliwal, 2018). A promising method to reduce the whitefly population and therefore chilli leaf curl virus disease is to understand the resistance mechanisms of chilli to whitefly and explore these traits for their potential in breeding resistant cultivars (Moshe and Michael, 2002).

It is known that whiteflies have an affinity for some particular genotypes as compared to others and this has resulted in some increased susceptibility to chilli leaf curl virus diease of some hybrids under open field conditions. The symptomless reaction of genotypes can be due to the non-preference of whiteflies (Banerjee and Kalloo, 1987), and may not be due to begomovirus resistance. Several sources of whitefly resistance have been identified in chilli (**Table 2** **).** Morphological resistance factors physically hinder the movement of the insect on the plant, and more specifically, interfere with the mechanisms of host selection, ingestion, digestion of plant material, mating and oviposition. Plant resistance to whiteflies has shown to be mediated by morphological characteristics of the leaf surface, such as trichome density, presence of glandular trichomes, and cuticle thickness (Jindal et al., 2008; Chermenskaya et al., 2009; Firdaus et al., 2011). The presence of thick cuticles was correlated with resistance to whitefly in chilli. Thick cuticle in combination with dense trichomes likely inhibits the insect style from reaching the phloem (Firdaus et al., 2011). The presence of glandular trichomes reduces the whitefly population and their nymphal density (Yadav et al., 2020). Rodríguez-Leal et al. (2017) reported high mortality of whiteflies on leaves with a high density of glandular trichomes due to the secretion of chemical compounds such as acyl sugars. However, in *C*. *annuum* accession PBC 535, *C*. *baccatum* accession No. 1553 and *C*. *frutescens* cultivar Tabasco, which all had glandular trichomes, were susceptible to whitefly, whereas the *C*. *annuum* accession CM331, which has only non-glandular trichomes, was highly resistant (Firdaus et al., 2011). The contrasting observations that glandular trichomes may and may not contribute to whitefly resistance in chilli indicate the diversity and complexity of resistance mechanisms to whiteflies based on trichome architecture, and shows the need for further investigations in this area.

**Table 2:** Sources of resistance in *Capsicum* spp. to whitefly, a vector of Begomovirus.

Genotypes	Resistance traits	Reference
*C. annuum* cv. Tabaquero, Amaxito	Antibiosis	Latournerie et al., 2015
California Wonder and Yolo Wonder	Resistant due to Glabrous leaves with thick cuticle	Firdaus, 2012
CA 9, CA28 and ACC 05	Strong antixenotic and antibiotic	Jeevanandham et al., 2018
IHR 4283, IHR 4329, IHR 4300, IHR 4321 and IHR 4338	Antibiosis	Yadav et al., 2020
IAC-1544, IAC-1545 and IAC-1579	Antibiosis	Pantoja et al., 2018
Qianhong, zhongjiao, hangjiao, zhonghuahong	Antixenosis	Jiao et al., 2018
CM331, Seranno and California Wonder 300	Trichome density and thick cuticle	Firdaus et al., 2011
Blanco, Bolita, and Pico Paloma	Antibiosis	Ballina et al., 2013
Aleppo	Antixenosis	Al-Aloosi et al., 2020

Color of the plant may also play an important role in protecting against attraction and ovipositional choice of whitefly (Hasanuzzaman et al., 2016). The purple-colored chilli population with anthocyanin accumulation are resistant to whitefly (Cheng et al., 2018), and to thrips and mites under field conditions (unpublished). The PepYLCThV resistant breeding line 9852-123, which was inoculated using augmented whitefly infestation, reported by Barchenger et al. (2019), was also purple; however, they were able to isolate viral DNA present in the leaves, indicating host resistance to the virus, and not whitefly.

Biochemical compounds influence insect feeding behavior, reproductive ability and host plant preference. These compounds include direct defenses mediated by plant toxins and indirect defenses, mediated by phenolic compounds, alkaloids, and terpenoids (Mithofer and Maffei, 2017). Plant secondary metabolites such as methyl-ketones and derivatives of sesquiterpene carboxylic acid can have negative effects on insect population development, as they can act as an attractant, repellent or antibiotic substance (Eigenbrode et al., 1996; Chermenskaya et al., 2009). Host resistance in chilli is positively correlated with phenol levels and peroxidase and polyphenol oxidase activity (Bhonwong et al., 2009; Jabeen et al., 2009; Mondal et al., 2013). Polyphenol oxidases and thionins were shown to be involved in maintaining the basal defense against fungi, bacteria, and viruses (Taranto et al., 2017), and ChiLCV resistant lines had higher levels of polyphenol oxidase and peroxidases activity than susceptible lines (Rai et al., 2010; Kushwaha et al., 2015; Manisha et al., 2020). The role of leaf characteristics such as the biochemical composition, metabolites, nutritional value, and defense related enzyme activity in whitefly resistance are not well studied. There is a need for an improved understanding of biochemical changes that affect virus-vector-host plant interactions. Genetic analysis of the genotypes/cultivars can assist in identifying the candidate genes implicated in resistance for being used in developing cultivars resistant against whiteflies. Furthermore, monitoring whitefly behavior in reaction to different biochemical compounds, such as feeding preference and oviposition, might provide insights into resistance mechanisms and aid in the development of effective management strategies.

### Resistance inheritance

Resistance breeding against biotic stresses remains a top priority in modern breeding programs (Miedaner, 2016). The basic steps in begomovirus resistance breeding include screening of germplasm, identifying the resistant sources, and then movement of resistance into adapted backgrounds (Thakur et al., 2018). The choice of the breeding method required to move resistance into adapted material is highly dependent on trait inheritance patterns (monogenic, oligogenic, or polygenic) (Siddique et al., 2022). Furthermore, inheritance patterns can provide a basis for experiments to understand the genetic mechanisms of host resistance and the development of molecular markers associated with resistance.

Deciphering the gene interactions among the loci for resistance/susceptibility in the plant to the corresponding virulence/avirulence in their pathogen is requisite in a resistance breeding program. While plants often have NB-LRR protein-based immunity to viruses, the antiviral mechanism mediated by RNA silencing is a more common mechanism (Voinnet, 2001). Antiviral RNA interference is the first layer of defense and the resistant genes can be considered as the second layer of defense against viruses. Virus resistance inheritance can be divided into two forms of dominant and recessive resistance (Kang et al., 2005). More than 80% of plant viral resistance loci are monogenically inherited and most of them have a recessive virus resistance locus (Truniger and Aranda, 2009), with a high level of strain and species specificity. Plant viruses use proteins, called host factors, for completion of their life cycles (Nagy and Pogany, 2011). The recessive virus resistance concept was derived from these host factors for viral infection (Truniger and Aranda, 2009). Mutations or deletions in the host factors can confer a durable virus resistance, called recessive resistance (Truniger and Aranda, 2009). Dominant R genes can be grouped into two classes, those encoding NB-LRRs and non-NB-LRRs (Gururani et al., 2012). The major class of R genes encode NB-LRR motifs with three domains that are responsible for interaction with other R proteins genes, are involved in indirect pathogens recognition (Collier and Moffett, 2009) and induction of resistance responses (Lukasik and Takken, 2009; Slootweg et al., 2010). Zarate et al. (2017) reported that host-defense responses triggered by some begomoviruses also trigger the salicylic acid pathway. Whitefly also plays a role in amending the gene expression of defense pathways (salicylic acid and jasmonic acid/ethylene pathways) (Jose Trinidad Ascencio-Ibanez et al., 2008) There is a considerable body of literature regarding the expression *Capsicum annuum* pathogenesis related (*CaPR*) genes in chilli plants when infested with whitefly (Yang et al., 2010). It has been reported there is upregulation of transcriptional expression of *CaPR1* gene for an SA- signalling pathway (Kim and Hwang, 2000), *CaPR4* gene for ET⁄ JA-responsive signalling pathway (Park et al., 2001), *CaPR10* gene for SA⁄ET⁄ JA-responsive signalling pathway (Park et al., 2004), and *Capsicum annuum* protease inhibitor II (*CaPIN II*) gene for JA-responsive signalling pathway (Shin et al., 2001; Song et al., 2005) during whitefly infestation. Zhang et al. (2012) demonstrated the Suppression of jasmonic acid mediated proteins in tobacco by βC1encoded in the beta-satellite of TYLCCNV.

The difficulty of introgression of a trait from some accessions (field-collected wild relatives) into commercial cultivars depends on the genetic complexity of the trait. The inheritance pattern depends upon the resistant source and also on the pathogen. Monogenic recessive inheritance of leaf curl virus resistance has been widely reported (Bal et al., 1995; Kumar et al., 2009; Rai et al., 2010; Anandhi and Khader, 2011; Rai et al., 2014; Koeda et al., 2021; Siddique et al., 2022). Inheritance of resistance in BG3821 appears to be controlled by two genes with duplicate recessive epistatic action (Garcia-Neria and Rivera-Bustamante, 2011garcia). In contrast, Sran et al. (2023) and Thakur et al. (2019) proposed the monogenic dominant resistance in a resistant source S-343 through artificial whitefly inoculation screening. Sharma et al. (2015) confirmed the 3:1 ratio segregation of the resistance gene in the T_1_ generation of transgenic chilli (cv. Kasi Anmol) through PCR analysis. The variation in gene inheritance patterns may be due to multiple factors, including the species/strain of the virus, the inoculation technique used, the rating system employed, environmental factors, in addition to different sources of resistance being used.

The durability of R genes depends upon the viral population dynamics, changes in pathogenicity and frequency of virulent isolates (Kang et al., 2005). Understanding the genetic basis of leaf curl virus resistance in chilli is key to monitoring and managing resistance (Preston and Mallory-Smith, 2001). Recessive resistance may be more durable than dominant resistance in theory (Fraser, 1990). However, despite extensive studies showing recessive gene action for resistance, breeding programs have not produced durable resistant commercial varieties and those that have been produced are quickly overcome. Moreover, few studies have focussed on the characterization of virus resistance genes. Therefore, there is a great need to study the mode of inheritance and gene action for other sources of resistance followed by characterisation of the resistance genes. The available reports on the inheritance of chilli leaf curl virus resistance are summarized in **Table 3**.

**Table 3:** Summary of the studies on the inheritance patterns of begomovirus resistance in *Capsicum* spp. including resistant source, generation(s) evaluated, strain or species of the virus, and mode of inheritance identified.

	Susceptible parent	Resistant parent	Population	Genetics	Reference	Virus species
1.	MS 341	S-343	F_2_	Single dominant gene	Thakur et al., 2019	Tomato leaf curl Joydebpur virus (ToLCJV)
2.	PBC535 BS 35	Bhut Jolokia	F_1_, F_2_, BC_1_	Single recessive gene	Rai et al., 2014	ChiLCV- VNS (Chilli leaf curl virus- varanasi strian)
3.	Phule Mukta	DLSSel.10	F_1_, F_2_, BC_1_	Single recessive gene	Maurya et al., 2019	ChiLCV (Chilli leaf curl virus), ToLCNDV (Tomato leaf curl NewDelhi virus)
4.	Phule Mukta	WBC SEL 5	F_1_, F_2_, BC_1_	Single recessive gene	Mathur et al., 2019	ChiLCV (Chilli leaf curl virus)
5.	PBC535	Bhut Jolokia	F_1_, F_2_, BC_1_	Single recessive gene	Kumar et al., 2009	ChiLCV (Chilli leaf curl virus)
6.	Bhut Jolokia	PBC535	F_1_, F_2_, BC_1_	Major recessive genes with some minor genes	Rai et al., 2010	ChiLCV-VNS (Chilli leaf curl virus- varanasi strian)
7.	Punjab lal	LLS and Hungarian Sweet Yellow	F_1_, F_2_, BC_1_P_1_ BC_1_P_2_	Single recessive gene	Bal et al., 1995	NR
8.	NR	Kashi Anmol	T_1_	Single recessive gene	Sharma et al., 2015	ChiLCV (Chilli leaf curl virus)
9.	NR	BG3821	S_1_	Two genes with duplicate recessive epistatic	Garcia-Neria and Rivera-Bustamante, 2011	Pepper golden mosaic virus (PepGMV)
10	Jintianchaojiao	Shishigongjiao	F_1_, F_2_, BC1	Single dominant gene	Chen et al., 2016	TYLCV (Tomato yellow leaf curl virus)
11	IIHR4517 and IIHR4630	IHR3476	F1, F2, BC1P1, BC1P2	Single dominant gene	Yadav et al., 2022	ChiLCV-Rai (Chilli leaf curl virus- Raipur strian)
12	BaPep-4	BaPep-5	F2	Single recessive gene	Pohan et al., 2023	Pepper yellow leaf curl Aceh virus (PepYLCAV)

### Screening techniques:

An adequate and proficient protocol for germplasm screening is required to be successful in breeding for resistance to ChiLCV (Koeda et al., 2015). Natural field screening was often used in the early 1960s to identify sources of resistance based on disease occurrence and severity of chilli leaf curl virus disease (Aiswarya et al., 2019). The use of “hot spots”, which are locations with high disease pressure combined with strain or species characterization using molecular tools are key for successful natural screening of leaf curl virus. Field screening for pathogens is generally ineffective, as many plants avoid infection, even under extreme inoculation pressure (Vidavsky et al., 1998). Feeding of other sucking pests in the field that resemble leaf curl symptoms can confound data collection and accurate selection. Field screenings do not allow for control over factors such as whitefly-vector pressure, the severity of inoculation (which relates to the intensity of symptoms caused by a virus in a plant), the amount of viral inoculum (referring to the quantity or concentration of virus particles introduced during infection), and the plant’s age at the time of inoculation (Lapidopt, 2007). Furthermore, natural screening may lead to unsynchronized infection, resulting in erroneous data. Resistance displayed by certain lines cannot be inferred as real host resistance since certain lines can escape whitefly, leading to false positive selections so the breeding program will get congested with a large number of susceptible lines. It is advisable to screen the germplasm for multiple seasons to reduce false positives and ensure selected lines are resistant (Vidavsky et al., 1998); however, multiple season screening can also result in screening with multiple species of the virus, given the high diversity of *Begomovirus*, further reducing selection accuracy in field trails.

Whitefly transmission of plant viruses is a valuable means of screening plants for resistance to viruses, as it uses the same type of transmission that plants in the field (Polston and Capobianco, 2013), but with more control of the inoculum concentration and timing and reduces escapes. For whitefly-medicated screening, a period of inoculation feeding of the whiteflies to the target plant should be adequate to ensure effective inoculation, but minimal enough to reduce the direct damage caused by white flies. Non-viruliferous whiteflies collection can be maintained on non-host plants or cauliflower (*Brassica oleracea* var. *botrytis* L.) or brocolli (*Brassica oleracea* var. *italica* Plenk) plants. Rocha et al. (2012) maintained non-viruliferous whiteflies on collard green (*Brassica oleraceae* var. *acephala* DC.) and soybean (Glycine max) plants, in a greenhouse with insect-proof nets. To obtain precise results in screening Rocha et al. (2011) identified the whitefly species collected by using PCR-RFLP analysis and sequencing the *mtCOI* gene. The whiteflies were allowed to feed on leaf curl virus infected plants for about 24 hrs (acquisition access period). Test plants were then infested with the viruliferous whiteflies (approximately 15-20 per plant) at the 2-4 true leaf stage for 48 hrs (inoculum access period). As chilli is the non-preferred host for whiteflies, in free choice assay we cannot ensure vector infection on all the plants and in no choice essay, a single plant viruliferous whiteflies are introduced on a single plant (enclosed in a small bottle cage). Whitefly colony establishment and maintenance, to transmit chilli leaf curl viruses to test entries for screening have been successfully utilized (Kumar et al., 2011; Padhi et al., 2017; Srivastava et al., 2017; Sharma et al., 2018; Srinivas and Thomas, 2018; Thakur et al., 2018; Barchenger et al., 2019; Maurya et al., 2019; Manisha et al., 2020; Yadav et al., 2020). Graft inoculation has been used to screen for TYLCV-resistant plants with high transmission efficiency (Fargette et al., 1996). A benefit of graft inoculation is that it enables a test plant to be continually exposed to high levels of viral inoculum. It is the safest method for the maintenance of leaf curl virus but not a preferred option for screening as it is not a high throughput screening, labor intensive, and time consuming.

Agro-infiltration mediated screening used under controlled laboratory environments with small seedlings has been used to facilitate a precise resistance assessment in a short time and space. The potential of using *Agrobacterium* as a vector to generate plants with genes of interest has been acknowledged and duly exploited by researchers to understand the functions of the identified gene(s) (Gelvin, 2003). A leaf disc agroinoculation system was developed to differentiate between susceptible and resistant tomato genotypes to TYLCV infection (Czosnek et al., 1993). Sakata et al. (2008) constructed partial tandem repeats of PepYLCIV DNA A and B, cloned them into a binary pGreenII vector and successfully agro inoculated *Nicotiana benthamiana* L. and *C. annuum* for screening. Kumar et al. (2011) produced infectious clones comprising of partial tandem repeats of the viral genome (1Æ9-mer) and the betasatellite (1Æ7 -mer) into vector pCAMBIA-1300 and agroinoculated to chilli and *N*. *benthamiana*. Plants inoculated with viral clones alone, do not produce leaf curl symptoms, but after inoculating along with beta satellite distinctive leaf curling and stunting symptoms were detected, indicating the importance of betasatellite. Shafiq et al. (2010) had observed leaf curl symptoms in *C. annuum*. *cv* Loungi when inoculated with partial repeats of PepLCLV along with the DNA B of ToLCNDV. Agroinoculation screenings have been widely used or chilli leaf curl virus strain (Shahid et al., 2019), tomato yellow leaf curl Kanchanaburi virus strain (Koeda et al., 2020), and tomato yellow leaf curl virus (Verlaan et al., 2011). The use of agroinoculation in breeding programs has been questioned because it does not account for any the natural resistance that might exist in some wild *Solanum* accessions by bypassing the early steps of virus infection (Kheyr-Pour et al., 1994). Koeda et al. (2017) reported agroinoculation combined with subsequent grafting, provides a highly efficient method for introducing pepper yellow leaf curl indonesia Virus (PepYLCIV) into chilli plants. Chauhan et al. (2018) revealed that ChiLCV can be transmitted by sap and out of the three methods used (syringe, rubbing and immersion); syringe inoculation was found the most efficient method for sap transmission. Chilli peppers are highly recalcitrant in terms of *in vitro* regeneration and genetic transformation. Though the utilization of Agrobacterium-mediated transformation is prevalent in chilli peppers, its effectiveness depends on successful shoot regeneration and the genotype (Lee et al., 2004). Standardizing correct inoculation protocols permit a rapid, reliable and reproducible selection of begomovirus resistant accessions.

### Symptom severity scoring

Symptom severity scales need to be established as a part of leaf curl virus inoculation protocols. Susceptible controls included in the screen should ideally become infected and show the highest symptom severity. The variability in assay conditions and symptom scoring scale will lead to contradictory results, where different resistance levels were attributed to the same genetic material. The scoring scale (0–5 point scale) was developed by Joshi and Choudhary (1981) and Banerjee and Kalloo (1987) for leaf curl virus screening in tomato. Being a slow-growing crop as compared to tomato, the solicitation of the same scale is not appropriate at the nursery stage. Chilli takes longer time for symptom appearance and a breeder will have to wait longer to study disease related traits. Sharma et al. (2018) developed a disease severity scale for screening at nursery stage (4-6 leaf stage) under artificial conditions. Yadav et al., 2020 adopted 0-4 scale (immune, highly resistant, resistant, moderately resistant and susceptible) for screening. A scoring scale (0–6 point scale) developed and used by the World Vegetable Center is now widely deployed across Asia and Africa in a coordinated manner to ensure selection accuracy in multiple location screening experiments (**Table 4**).

**Table 4:** Symptom scaling for leaf curl virus screening.

S. No.	Symptom	% crop affected	Disease reaction
1	Plants appear visibly free of any symptoms	0	Symptomless
2	Mild vein thickening is observed on new leaves, Canopy growth and plant height are not affected, Mild inward curling of young leaves is observed	0-10	Resistant
3	Symptoms of curling are observed on young top shoots of every branch Lower leaves show clear vein thickening infected leaves appear to be of smaller size	11-30	Moderately tolerant
4	Leaf cupping and curling, typical vein thickening symptoms. Plant height is below normal, with branches having short internodes	30-60%	Moderately susceptible
5	Cupping of the leaf, shortening of internodes, vein thickening, and the plant becomes severely stunted	60-90%	Susceptible
6	Severe curling and cupping, stunted with a bushy appearance	90-100%	Highly susceptible

### Identification of resistance gene loci and molecular marker development

Given that sources of stable and durable resistance are rare, it is not surprising that the identification of loci and associated molecular markers contributing to leaf curl virus resistance in chilli are limited. In a recent study, Siddique et al. (2022) employed genotyping-by-sequencing-based QTL mapping to discover three QTLs, peplcv-1, peplcv-7, and peplcv-12 on chromosomes P1, P7, and P12 respectively. The researchers additionally developed markers (Chr7-LCV-7, Chr12-LCV-12) and confirmed their efficacy through validation in an F2 population and across various commercial varieties. Similarly, Koeda et al. (2021) discovered a codominant CAPS marker, S05_14208507, located on chromosome 5, designed for detecting pepper yellow leaf curl Indonesia virus (PepYLCIV). Additionally, Thakur et al. (2020) identified two molecular markers (CA516044 and PAU-LC-343-1) on chromosome 6, that were associated with Tomato leaf curl Joydebpur virus resistance in chilli. Compared to chilli, molecular markers have been extensively developed in tomato based on QTLs controlling resistance to tomato yellow leaf curl virus (TYLCV) (Kadirvel et al., 2013). Previous studies have emphasized the high genome synteny and collinearity among crops in the family Solanaceae (Grube et al., 2000). Among related host species, structural and functional conservation of R genes have been previously reported (Grube et al., 2000). A high level of synteny between the major *Phytophthora capsici* resistance locus of chilli and potato has been documented (Sandbrik et al., 2000; Thabuis et al., 2004) and integration of the *RB* gene from *S*. *bulbocastanum* into chilli via *Agrobacterium tumefaciens* mediated transformation conferred a high level of *P. capsici* resistance (Bagga et al., 2019). Orthologous genes of *C*. *annuum* proteins involved in the pepper-PepGMV recovery response were also found in tomato and potato, which suggested the conservation of the defense response pathway in different hosts (Zanardo et al., 2019). Genes conferring resistance to tobacco mosaic virus (TMV, *Tobamovirus*), cucumber mosaic virus (CMV, *Cucumovirus*), tomato spotted wilt virus (TSWV, *Tospovirus*) and members of *Potyvirus* in tomato and potato have been found to co-map in the homologous genomic region in chilli (Kim et al., 2017; Venkatesh et al., 2018). However, using the *Ty* loci from tomato, Manisha et al. (2017) was unable to identify genes in chilli that conferred resistance to begomovirus. Similarly, Manisha et al. (2017) performed Bulk Segregant Analysis (BSA) in F_2_ segregating populations derived from PM × DLS-Sel-10 and Anugraha × WBC-Sel 5 with 86 orthologous markers in the various *Ty* regions of tomato; however, none of the markers were linked to ChLCV resistance genes/QTLs.

To understand the host defense mechanism, transcriptomic profiling of an infected host can be an effective strategy. Gongora-Castillo et al. (2012) used transcriptome sequencing to compare the response on the transcriptome level in recovered and not recovered chilli leaves that were infected by the bipartite pepper golden mosaic virus (PepGMV). The authors found 309 differentially expressed genes (168 up-regulated and 141 down-regulated) that were associated with different cellular and physiological processes of the recovery process of the host after infection. Similarly, a comparative expression study between resistant and susceptible chilli leaf curl virus infected plants, demonstrated a up to 5-fold up-regulation of several NBS-LRR domain genes in resistant lines (Kushwaha et al., 2015). Also in a recent study, the expression of *Ca-NBS-LRR* genes was found to be higher in the ChiLCV resistant genotype DLS-Sel-10 than the susceptible cultivar Phule Mukta inoculated with (Manisha et al., 2020).

Polyphenol oxidase (PPO) has been found to assist in basal defense against fungi, bacteria, and viruses (Poiatti et al., 2009) and PPO transcript levels were elevated in the resistant chilli cultivar Punjab Lal, suggesting PPO could play a role in initiation of basal defence against ChiLCV infection (Kushwaha et al., 2015), or the upregulation of this gene reflects a general stress response to virus infection. Likewise, Kushwaha et al. (2019), investigated genes downregulated upon virus infection in chilli variety Punjab Lal by reverse suppression subtractive hybridization follow Based on an interaction map approximately 35% of all downregulated expressed sequence tags were homologous with genes that encode chloroplast proteins and 16% of the genes were predicted to be involved in biotic or abiotic stress response. However, no QTL studies tracking LCD resistance in pepper mapping populations or germplasm panels have been reported yet.

### Identification of Begomovirus resistant accessions

Given the difficulty and low-throughput of single isolate inoculation and the preponderance of multiple begomovirus to occur in a single field, the most common strategy employed by researchers to identify resistant accessions or screen segregating populations by growing the plants under open-field conditions at disease hot-spots (**Table 1**). Open field screening is an inexpensive and relatively easy technique, but it may lead to mixed infections from diverse viral species and genera and mixed infection which makes it difficult to correctly identify resistance sources (Kenyon et al., 2014a; Jo et al., 2017). Even after several years of screening efforts, no chilli accessions with broad resistance to diverse species and strains of begomovirus has been identified, indicating that such broad resistance may not exist, or it is at least extremely rare in the available germplasm. Similar to the efforts to generate *Ty* resistant materials in tomato, systematic research needs to be carried out to identify strain specific resistance mechanisms in chilli. This will require the regular monitoring of the pathogen population in the hot spots for the disease and screening of accessions for the most prominent and emerging strains of begomovirus using single isolate inoculation techniques. As has been described above, begomoviruses have high levels of genetic recombination and mutation as well as have interactions with other species and strains. Therefore, such a monitoring effort will require local, regional and international cooperation on an annual or seasonal basis. Once the predominant strains have been identified, single isolate associations will need to be done across a large number of accessions. There are numerous methods to conduct single-isolate inoculation including grafting, agroinfiltration, ballistic bombardment, augmented inoculation by viruliferous whitefly (Barchenger et al., 2019) among others. However, the common requirement of all these inoculation methods is the exclusion of the variability introduced by the vector, typically through isolation of the plants along with pest monitoring and appropriate pesticide application, to eliminate the possibility of co- or mixed-infection.

Although no systematic studies have been done to evaluate the effectiveness of the various inoculation methods and associations with disease incidence and severity in open field conditions, we prefer the augmented inoculation by viruliferous whitefly method. This method is relatively fast and less expensive as compared to the grafting method, but does introduce the possibility of selection for lack of vector preference based on plant morphology. Lack of preference by the whitefly vector would be useful in breeding for durable resistance, but it makes the identification of host-resistance genes difficult. The agroinfiltration method eliminates the possibility of confounding host resistance to the vector and the virus, but is more difficult, expensive and requires the ability to develop artificial constructs of the virus, which is not possible in many locations where begomovirus is a serious problem. Due to the presence of strain-specific resistance, gene pyramiding has been used as an effective approach to achieve durable resistance with high accuracy in tomato. Many TYLCV resistance genes (*Ty-1/Ty-3, Ty-2, Ty-4, ty-5, ty-6*) have been identified, well characterized and mapped in tomato. Prabhandakavi et al. (2021) developed a tomato leaf curl virus (ToLCV) resistant commercial tomato hybrid, ‘JKHT1’, through pyramiding of *Ty-1*/*Ty-3*, *Ty-2*, *ty-5*, and *ty-6* genes with help of marker-assisted backcross breeding. Similarly, Hanson et al. (2016) described the need to combine *Ty2* and *Ty3* to confer moderate levels of resistance to tomato yellow leaf curl Taiwan virus (TYLTwV) and tomato yellow leaf curl Thailand virus (TYLCThV). Strain specific screening will allow the identification of resistant accessions, and after development of segregating populations, the mapping of the putative resistance genes and the design of associated molecular markers to tag strain-specific resistance or partial resistance genes for breeding and gene pyramiding. It is likely that the use of strain specific resistance will not immediately result in the development of highly resistant cultivars; however, by understanding the mechanisms of resistance against single strains or moderate levels of resistance. Strain specific resistance gene characterization and gene pyramiding can be deployed for the development of durable host resistance against begomovirus in chilli, but mapping the possibly low levels of resistance may hinder resistance mapping and subsequent gene pyramiding. In this situation, it may be necessary to identify multiple sources of moderate levels of resistance and perform breeding using recurrent selection using single strain screening.

The goal of a recurrent selection program is to increase the proportion of a particular trait in a population, and would first require a basic study on the relatedness of the sources of moderate resistance in the program. Ideally, the donors of resistance should be distantly related to increase the probability that various different resistance genes are available for recurrent selection. After the first screening, selections are made and hybridized in reciprocal, the segregating populations are then screened again using the single isolate approach. This process of inoculation, selection, and hybridization is repeated until a number of lines with higher levels of resistance are identified. Recurrent selection breeding could be done for several different strains until resistance is found for most of the predominant strains in a region. At that point, mapping populations can be developed, QTLs identified, and associated molecular markers validated, which would facilitate gene pyramiding. Using single isolate screening techniques, sources of resistance to PepGMV, PHV, PepYLCThV, and tomato leaf curl Joydebpur virus (ToLCJoV) have been identified (Garcia-Neria and Rivera-Bustamante, 2011; Barchenger et al., 2019; Thakur et al., 2019), providing a basis to initiate this work, but there remains much to do.

An alternative approach could be to generate a multi-parent advanced generation intercross (MAGIC) population derived from different parents that showed resistance to LCD at different disease hot spots. Resultant inbred lines could be selected for resistance to multiple viruses at disease hotspots. The advantage of the MAGIC population approach is that only one round of screening at disease hot spots is necessary to identify resistant materials and resistance genes can be directly mapped in the MAGIC population without additional crosses.

### Molecular breeding approaches

#### Pathogen derived resistance

PDR has potential for identification of begomoviruses resistance transforming a susceptible host by incorporating a sequence of genome derived from the pathogen. For plant viruses, the concept of PDR was first validated with the creation of tobacco plants expressing the coat protein gene of tobacco mosaic virus (TMV; *Tobamovirus*) and exhibiting resistance to infection by TMV (Abel et al., 1986). Viral genes have been widely used in the development of transgenic-resistant plants (pathogen-derived resistance) and have been effective in different pathosystems (Goldbach et al., 2003). Likely the most famous use of PDR is for papaya ringspot virus (PRSV; *Potyvirus*) in papaya and widely commercialized in the US (Gonsalves, 1998). Several studies indicated that the antiviral strategies such as DNA methylation, ubiquitination mediated defense and activation of gene silencing machinery can be effective against begomoviruses (Marino et al., 2012; Sahu et al., 2014). Applying the concept of PDR provides unique opportunities for developing begomovirus resistant chilli and implementing efficient and environmentally sound management approaches to mitigate the impact of viral diseases. The prospects of further advancing this innovative technology for practical control of viral diseases are very promising; however, consumer acceptance of GM crops in some of the largest producing countries with the biggest ChiLCV problem is still limited. It is possible that begomovirus resistant chilli using PDR could be developed and released in certain countries such as Bangladesh and China, where higher levels of consumer acceptance of GM crops exist.

#### Gene editing

Gene editing can be achieved through site specific mutagenesis using zinc finger nucleases (ZFNs), transcription activator-like effector nucleases (TALENs), and clustered regularly interspaced short palindromic repeats/Cas9 (CRISPR/Cas9). CRISPR/Cas9 has evolved as an effective and user-friendly tool for precise and predictable targeted mutations, mostly small deletions. Inducing begomovirus resistance in chilli through mutagenesis of host factors in the plant is theoretically an option, but it would require knowledge about which genes to target in which manner. Without this knowledge, for making plants resistant to LCD, CRISPR/Cas9 only can be targeted to viral DNA. Roy et al. (2019) designed nine-duplex and two-triplex CRISPR-Cas9 constructs to target the chilli leaf curl virus (ChiLCV) genome after virus infection in tobacco. They observed three of the designed constructs (gRNA5 + 4, gRNA5 + 2 and gRNA1 + 2) were effective in reducing the ChiLCV viral titer and symptom severity. Similarly, resistance to tomato yellow leaf curl virus (TYLCV) and bean yellow dwarf virus (BeYDV, Mastrevirus) in tobacco was enhanced by knock-out of the coat protein gene of geminiviruses through the application of CRISPR-Cas9-mediated mutagenesis (Baltes et al., 2015; Ghorbani et al., 2020). However, the use of CRISPR/Cas9 to target viral ORFs may result in new viral variants, which could lead to various levels of viral escape events, increasing the risk that instead of controlling the virus, it generates new mutant viruses that could spread to other plants. However, using a multiplexed guide RNA (gRNA)-dependent CRISPR-Cas9 method that targets the viral genome at multiple sites simultaneously, reduces the risk of generating mutants and can successfully eliminate ChiLCV from infected plants (Roy et al., 2019). Unlike genetic modification via *Agrobacterium*, plants originating from the use of CRISPR-Cas9 mediated gene editing that do not contain any foreign DNA are not considered GM crops in countries with a product-based GMO law, like in the US, Argentina or Japan. However, to be effective against virus infection, CRISPR/Cas9 needs to be present and active in the plants, which means that such plants carry foreign DNA and therefore are considered to be GMO organisms according to the Cartagena protocol on biosafety. Furthermore, like for GMO production, for most CRISPR/Cas9 applications, a stable regeneration system is required. As reviewed by Barchenger et al. (2018), transformation in chilli is highly genotype-specific and different protocols are required with different accessions.

#### Gene silencing

RNA interference (RNAi) is a biological process in which RNA molecules inhibit gene expression or translation, by neutralizing targeted mRNA molecules. The exploitation of RNAi using various viral genes (REP, CP, V2, etc.) may help in controlling the disease. Sharma et al. (2015) generated transgenic chilli, cultivar Kasi Anmol, and tobacco (*Nicotiana benthamiana*) plants which had resistance to the begomoviruses using RNAi mediated gene silencing using two different hairpin RNAi TR1 (TR1-15 and TR1-8) and TR4 (TR4-1and TR4-2) constructs. RNAi method was used in tomatoes to develop resistance against multiple begomoviruses (Chen et al., 2016) and CLCuD in cotton (Sattar et al., 2013). Sharma and Prasad (2020) engineered transgenic plants by expressing artificial microRNAs (amiRNAs) that provide defense against the AC1 gene of tomato leaf curl New Delhi virus (ToLCNDV). In chilli, resistance to the Kor strain of cucumber mosaic virus (CMV-Kor, *Cucumovirus*) and pepper mild mottle virus (PMMoV; *Tobamovirus*) has been developed using sense gene induced posttranscriptional gene silencing (S-PTGS) and co-expression of the coat proteins (CPs) of CMV-Kor and tomato mosaic virus (ToMV; *Tobamovirus*) (Shin et al., 2002). siRNA mediated resistance was reported in tobacco against tomato yellow leaf curl virus-Oman (TYLCVOM) (Ammara et al., 2015), pepper golden mosaic virus (PepGMV) (Medina-Hernández et al., 2013), chilli leaf curl virus (ChiLCV), tomato leaf curl New Delhi virus (ToLCNDV), and chilli leaf curl Vellanad virus (ChiLCVeV) (Sharma et al., 2015) and in tomato against tomato yellow leaf curl virus (TYLCV) (Fuentes et al., 2016). *CchGLP* is a gene that encodes Germin-like proteins (GLPs), which play a crucial role in plant defense against viral infections. This gene was discovered in *Capsicum chinense* (Jacq.) accession BG-3821, which exhibits resistance to geminivirus infection. Through the Virus-induced gene silencing technique, the *CchGLP* gene was suppressed in BG-3821, resulting in susceptibility to geminivirus in BG-3821 (Mejía-Teniente et al., 2015). Additionally, when *CchGLP* was introduced into geminivirus-susceptible *Nicotiana tabacum xanthi* nc plants via transgenic approach, it led to the amelioration of symptoms in transgeni plants in comparison to non-transgenic (Guevara-Olvera et al., 2012).

Gene silencing by artificial microRNAs (amiRNA) facilitated gene regulation at the post- transcriptional or translational level (Bartel, 2004). They regulate gene expression by degradation or translation repression of target mRNAs (Meyers and Axtell, 2019). This approach has been adapted for precise silencing of viral genomes in plants. Sharma and Prasad (2020) developed an artificial microRNA (amiRNA) based system against ATP binding domain of AC1 using endogenous precursor, miR319a, which provided defense against ToLCNDV in tomato. Overexpression of amiRNA to various virus genes can result in tolerance against viral infection (Petchthai et al., 2018). The amiRNA approach has been deployed to develop resistance against cotton leaf curl burewala virus (CLCuD) in cotton and jatropha leaf curl gujarat virus (JLCuGV) in tobacco (Swetha et al., 2018; More et al., 2021). Recently, Mishra et al. (2020b) predicted potential mir-miRNAs through in silico analysis, and found some with high sequence similarity to the V1 coat protein and C1 (Rep) genes of ChiLCV, which could be used in the future as a target in amiRNA in chilli. These studies help in understanding the viral gene expression and regulation by host miRNAs that could pave a way into design strategies for defense against ChiLCV infection.

Spray-induced gene silencing (SIGS) is a non-transformative strategy for plant protection involving the spraying of double stranded RNA (dsRNA) or small interfering RNA (siRNA), which target pathogen genes on plant tissues (Worrall et al., 2019). The dsRNA targeting a pathogen gene is sprayed onto the plant’s surfaces. The pathogen directly takes the dsRNAs up and induces the pathogen RNAi machinery, or the host plant takes dsRNAs up first and induces the plant RNAi machinery, and then dsRNAs or siRNAs are transferred into pathogenic cells and induce the pathogen’s RNAi machinery. Thus, this approach silences pathogen’s genes without introducing heritable modifications into the plant genome (Koch et al., 2016; Wang and Jin, 2017). Liu et al. (2020) have combined artificial microRNA (amiRNA)-mediated silencing technology and clay nanosheet mediated delivery by spraying for TYLCV infection in tomato plants. Three plant expression vectors expressing pre-amiRNAs were constructed, and recombinant plasmid DNAs (pDNAs) were loaded onto layered double hydroxide (LDH) clay nanosheets. The LDH nanosheets coated with pDNAs were sprayed onto plants infected by TYLCV, and both the disease severity and TYLCV viral concentration in sprayed plants was significantly decreased. These findings show that LDH nanosheets loaded with amiRNAs expression pDNAs can be a promising method for begomovirus control. How far this experimental approach is cost effective and can be applied by smallholder farmers needs still to be demonstrated.

### Mutagenesis

Mutagenesis has been previously applied to create variation for traits that are not present in the existing germplasm, as well as for studies of functional genomics in many crops. Mutation breeding is most effective when the loss of function of a gene result in a desirable phenotype. There are several types of mutagens that have been applied for this purpose, including chemical mutagens to induce specific types of base substitutions at a high frequency for functional genomics and the application of reverse genetics studies (Stephenson et al., 2010; Okabe et al., 2011). Radiation that induces a broad range of mutations such as point mutations, large insertions and deletions, chromosomal aberrations, and rearrangement, resulting in a higher probability of loss-of-function mutations, while maintaining overall lower mutation rates, compared to chemical mutagens (Shirasawa et al., 2016). In chilli, multiple studies have used mutant populations obtained by EMS mutagenesis (Bosland, 2002; Hwang et al., 2014; Siddique et al., 2020) and gamma radiation (Jo et al., 2016).

Mutagenesis has resulted in the identification of target specific genes and help to identify resistance genes and their function (Abe et al., 2012; Steuernagel et al., 2016). Normal recessive *Potyvirus* resistance in chilli is conferred by mutations in the eukaryotic translation initiation factor genes *eIF4E* or *eIFiso4E*, which impede interaction of these genes with the viral VPg protein (Sanfacon, 2015). Recessive resistance to chilli veinal mottle virus (ChiVMV; *Potyvirus*) is achieved through a double mutation of *eIF4E* (*pvr1*) and *eIFiso4E* (*pvr6*) translation factors (Hwang et al., 2009). In 2019, KeyGene scientists reported a loss of susceptibility gene to begomovirus in a sweet pepper line. The minor mutation was reported to be in a DTP gene that has high sequence similarity to Pelota (Pelo), which is encoded by the *ty5* locus in tomato. These findings have recently been validated by Koeda et al. (2021), who found a recessive resistance gene (*pepy-1*) in the resistant line Perintis that encoded the Pelota protein. More recently, Manzila and Priyatno (2020) identified 15 pepper yellow leaf curl virus (PepYLCV)-resistant mutant lines in the M3 generation of ‘Gelora’ by EMS-induced mutation. While there is strong evidence supporting the use of mutagenesis, especially EMS, for the identification of resistance to begomovirus, the use of gamma radiation has not been widely used. Gamma radiation results in overall lower mutation rates, while still resulting in a broad range of mutation, making it a promising tool that warrants further study. In mungbean, begomovirus resistant lines with good agronomic traits were developed by radiation breeding (Ali et al., 1997). Unlike several of the other strategies discussed here, plants developed using mutagens are not restricted for use in the ways that many highly targeted genetic transformation technologies are, increasing the usefulness of this strategy to develop resistant varieties.

### Pangenomics

Access to high quality reference genomes plays an important role in research and genomics assisted breeding. Understanding how specific genes and variants contribute to quantitative traits such as broad spectrum resistance to begomoviruses is necessary. Short-read sequencing has certainly accelerated the discovery of genetic variants, especially SNPs and indels (Nimmakayala et al., 2016; Siddique et al., 2019). However, it is known that structural variations, such as large insertions or deletions, duplications, and chromosomal rearrangements affect traits of agricultural importance (Lye and Purugganan, 2019). We also find that standard short read genotyping-by-sequencing (GBS) results in a higher than expected level of heterozygosity. We recently conducted GBS in an F11 RIL population and found that approximately 35% of the markers were heterozygous, which is 700 times higher than expected (>0.05%) (Unpublished data). The high level of heterozygosity limits the SNP-calling accuracy and ultimately reduces the effectiveness of QTL mapping. Based on the thorough bioinformatic analysis, we hypothesize that the high level of heterozygosity was not due to read quality, transposable elements (repeat sequences), or heterochromatin (low recombination), but due to missing data in the reference genome.

Qin et al. (2014) suggested that transpositions played a role in the domestication of *Capsicum*, but this area of research has not been further explored. To overcome the challenges associated with repetitive sequences, Ou et al. (2018) established a first Pepper Pan-Genome analysis, based on 10x coverage short-read re-sequencing data of 383 domesticated *Capsicum* accessions projected against the Zunla-1 reference genome limiting the study in the power to detect reliably structural variation (SV), especially large SV. Recently, Shirasawa et al., 2023 employed optical and genetic mapping to create a chromosome-scale genome assembly for the ‘Takanotsume’ chili line, revealing nucleotide sequence variations, chromosomal structural rearrangements, and transposon-insertion polymorphisms through comparative genomics within the Capsicum species. Similarly, Lee et al., 2022 condcuted a pan-genome analysis and reported significant large structural variants (SVs) in the pepper genome, including presence-absence variants (PAVs), inversions, and copy-number variants (CNVs). Notably, this analysis highlighted the presence of PAVs associated with valuable traits such as potyvirus resistance along with other traits in chilli peppers, offering potential insights for genetic analysis and genome-assisted breeding strategies to enhance pepper improvement. In tomato, Alonge et al., 2020 developed a pan-genome using long read nanopore sequencing and found that structural variation affected hundreds of genes with subtle to significant effects, resulting in changes in gene dosage and expression levels for quantitative traits such as fruit flavor, size, and production and important harvesting traits. Prominent examples for the impact of SV on (epigenetic) gene regulation in crop production were given in oil palm (Ong-Abdullah et al., 2015) and tomato (Rodríguez-Leal et al., 2017) underpinning the importance in agriculture for the detailed understanding of genic but also intergenic SV in cultivated and wild germplasm (Eshed and Lippman, 2019). The role of SV underlying important quantitative traits in chilli is underexplored. Chilli provides an interesting model to study structural variation due to the high proportion of repetitive sequences and associated large genome, hence there is an urgent need of establishing the required genomic resources to better understand resistance to begomovirus as well as many other traits.

## Conclusions

In conclusion, chilli leaf curl virus disease and the associated begomoviruses continue to pose a significant threat to chilli production worldwide. Developing sustainable and effective control measures to mitigate yield losses due to LCD is crucial. In principle, there are two pathways towards leaf curl virus-resistant chilli breeding cultivars that are resistant to the whitefly vector and developing varieties that resist begomovirus. In the long term, combining both approaches toward resistance is the preferred strategy. Several whitefly-resistant chilli lines have been identified, which rely on morphological and chemical defenses. Although resistance against specific Begomovirus strains is available, breeding for broad resistance against multiple viral strains remains a challenge. Moreover, the inability to diagnose begomovirus infection at an early stage is a barrier to illness management. The way forward: 1) strengthening resistance against the vector, 2) identifying germplasm resources with resistance against various begomovirus strains, and subsequent pyramiding of the resistance through recurrent selection and through MAGIC populations could be an effective strategy. The use of integrated pest management techniques that incorporate genetic resistance, cultural practices, and chemical control should be incentivized for the long-term management of the leaf curl virus. Finally, more research is required to comprehend the intricate interactions between the whitefly vector, begomovirus, chilli plants, and the environment. Advances in molecular biology, genomics, transcriptomics, and bioinformatics can provide new insights into the mechanisms of host-pathogen-vector interactions and hasten the development of effective and long-term management strategies for chilli begomovirus diseases like chilli leaf curl virus disease. To address the challenges of begomoviruses and ensure sustainable chilli production in the future, researchers, breeders, farmers, and policymakers must work together.

## Author contributions

DB and MK conceptualized the study. DB, MK, RS, and HP reviewed and edited the manuscript. All authors contributed to the article and approved the submitted version.
